# Three Anomalies I Have Met with in Castration1Read before the Fifteenth Annual Meeting of the Iowa State Veterinary Medical Association, January 14 and 15, 1903.

**Published:** 1903-03

**Authors:** J. R. Sanders

**Affiliations:** Corydon, Iowa


### REPORTS OF CASES.

### THREE ANOMALIES I HAVE MET WITH IN CASTRATION.[1]

By J. R. Sanders, M.D.C.,
Corydon, Iowa.

In reporting these cases I will dispense with a description of the manner of casting and the mode of disinfecting. I will say, however, that in each case due regard was had for antisepsis.

Case I. Hermaphrodite, belonging to Mr. Sager, three miles southeast of Promise City, Iowa. This gentleman informed me that he had a mare that would behave like a stallion when in company with mares that were in heat, and, owing to her bad disposition, he was compelled to keep her to herself. May 16, 1902, I had occasion to be in his vicinity. Mr. S., being informed by telephone, soon arrived with the mare, which I observed was a well-formed, muscular animal of the Percheron breed, rather large, dark, iron-gray, three years old, and in very good flesh.

Ordinarily one would not suspect another sex in disguise. Closer inspection, however, showed that there was about an equal tendency toward either sex. The udder was large and pendent, resembling a gland that had once been active. There was no commissure in what appeared to be a queerly-formed vulva, which rounded off inferiorly to a rudimentary penis, which was pierced with the urethral opening. The testicles could not be located by digital examination, consequently I decided they must be intra-abdominal. The owner requested me to find them, if possible, regardless of danger, as the animal was not worth much as it was. For the sake of being brief I will only say that the animal was cast and operated upon, both testicles being found inside the abdominal cavity close to the abdominal ring, and brought to the surface and removed. A good recovery followed.

Case II. Cryptorchid mule. I was called to our county farm April 26, 1898, to castrate a ridgeling mule two years old, medium weight, and in good condition. The left testicle had been removed the year before. The mule was cast and held upon his back by two men. After making an incision opposite the median line the hand endeavored to follow the natural course of the canal. The perito-

[1] Read before the Fifteenth Annual Meeting of the Iowa State Veterinary Medical Association, January 14 and 15, 1903.

neum was opened close to the abdominal ring. Two fingers inside the vas deferens was found without any trouble and brought out. The testicle would not follow. Holding the vas in one hand, I again searched inside the peritoneum as far as two fingers could reach. Not finding the testicle, I thought I would try taking off the vas, which was unusually large. This did not prove a success, for in about two weeks the warden informed me that the mule was as bad as ever, and had to be stalled on account of his amorous disposition. He thought the donkey must have had three seeds. Taking his advice I departed at once to remove, if possible, the other seed. Securing the animal as before, I gained the abdominal cavity close to the old wound. With the whole hand inside I soon discovered, about half-way to the renal organs, a mass about the size and shape of a large cocoanut. This was pulled loose from its attachment and, with considerable traction, brought through the opening. This proved to be a testicle with red surface, indicating recent inflammation, the centre being filled with dark-colored matter, fluid in consistency, which probably made up four-fifths of the entire bulk. Why this gland in its diseased condition influenced its possessor is a mystery. A good recovery resulted.

Case III. Cryptorchid mule, two years old, rather large and in good condition; right testicle in scrotum, left intra-abdominal. Operation took place on the owner's premises May 28, 1901. The animal was cast and held in position by assistants, and the incision made about one and one-half inches to left of the raphé. The canal was followed as near as possible, and two fingers entered the abdominal cavity near the ring. After about five minutes' search I located an unusually large vas, which was brought to the surface. Traction could not induce the testicle to follow. Thinking I might have a case similar to No. II., I began to search the abdominal cavity with the whole hand. By following the vas in for several inches I could plainly make out an irregular mass, with an osseous formation in the interior. Near this hard part I could feel an attachment to something, presumably the mesentery. A little manipulation with the fingers caused its separation, and the supposed testicle brought to the outside and removed. This specimen I have preserved in alcohol and have brought with me. Anyone thinking it worth while can inspect it. I have never investigated the hard substance. The other I am confident is testicular tissue.

Perhaps I should remark that after the mule regained his feet a loop of intestine dropped out and descended very near to his hocks. Knowing the mule to be of good disposition, as he was raised a pet,

I lay hold and suspended the bowel until he was recast, when it was returned and secured by bringing the incision together with two deep, strong sutures, which were removed in twenty-four hours. The owner informed me two weeks later that his mule never seemed any the worse by the operation.

### Discussion.

Dr. C. E. Stewart said that, although he has had a large experience in cryptorchid castration, he has never met with a cryptorchid mule.

Dr. Moyer said he castrated a cryptorchid mule in Illinois some years ago; also, that he met with an hermaphrodite equine, with external genitals resembling those of the female, from which he removed two hidden testicles.

